# Effects of Ocular Hypertension in the Visual System of Pigmented Mice

**DOI:** 10.1371/journal.pone.0121134

**Published:** 2015-03-26

**Authors:** Francisco J. Valiente-Soriano, Manuel Salinas-Navarro, Manuel Jiménez-López, Luis Alarcón-Martínez, Arturo Ortín-Martínez, José M. Bernal-Garro, Marcelino Avilés-Trigueros, Marta Agudo-Barriuso, María P. Villegas-Pérez, Manuel Vidal-Sanz

**Affiliations:** 1 Departamento de Oftalmología, Facultad de Medicina, Universidad de Murcia. 30.100 Murcia, Spain; 2 Instituto Murciano de Investigación Biosanitaria Virgen de la Arrixaca (IMIB-Arrixaca) 30.100 Murcia, Spain; NIH/NEI, UNITED STATES

## Abstract

To study the effects of ocular hypertension (OHT) on the visual system of C57BL/6 pigmented mice, the limbal and episcleral veins of the left eye were laser photocoagulated (LP). LP increased the intraocular pressure during the first five days (d), reaching basal values at 7d. To investigate the effect of OHT on the retinal ganglion cell (RGC) retrograde axonal transport, hydroxistilbamidine methanesulfonate (OHSt) was applied to both superior colliculi (SCi) and the retinas were dissected 2 or 4 weeks after LP. To determine RGC survival, these same retinas were immunoreacted against Brn3a (general RGC population) and melanopsin (intrinsically photosensitive RGCs, m^+^RGCs). To study whether OHT affected non-RGC neurons in the ganglion cell layer (GCL), RGCs were immunodetected with Brn3a and all GCL nuclei counterstained with DAPI in a group of animals examined 4 weeks post-LP. Innervation of the SCi was examined at 10 days, 8 or 14 weeks after LP with the orthogradely transported cholera toxin subunit-B. OHT resulted in diffuse and sectorial loss of OHSt+RGCs (50% at 2 weeks and 62% at 4 weeks) and in a comparable loss of Brn3a+RGCs at the same time intervals. m+RGCs decreased to 59% at 2 weeks and to 46% at 4 weeks, such loss was diffuse, did not parallel the sectorial loss of the general RGC population and was more severe in the superior-temporal retina. In the GCL, cell loss is selective for RGCs and does not affect other non-RGC neurons. The retinotectal innervation appeared significantly reduced at 10 days (55.7%) and did not progress further up to 14 weeks (46.6%). Thus, LP-induced OHT results in retrograde degeneration of RGCs and m+RGCs, as well as in the loss of CTB-labelled retinotectal terminals.

## Introduction

Glaucomatous optic neuropathies (GON) are a leading cause of blindness in the developed countries. One of the most important risk factors in GON is ocular hypertension (OHT), probably the only risk factor for which there are current therapeutic approaches [1]. Therefore OHT has attracted great interest among the scientific community [2,3]. There are several experimental models to induce and thus study ocular hypertension in adult mice such as: the episcleral vein occlusion [4] or the injection of polystyrene microbeads into the anterior chamber [5–7]. In addition, there are several congenic mice models of ocular hypertension including mice with a targeted type I collagen mutation [8] and the DBA/2J mice which develop a pigmentary glaucoma [9–15]. One of the most popular models involves laser cauterization of the episcleral and perilimbal veins in adult albino rats [16–21]. There are also several reports in adult albino [22–28] or pigmented [29–36] mice.

The present study further characterizes the effects of elevated intraocular pressure in adult pigmented mice; here we report the effect of laser photocoagulation (LP) of the perilimbar veins as well as of the episcleral vessels on the survival of the population of RGCs. Because experimental glaucoma has been shown to affect the circadian timing system [37,38] and several reports claim that intrinsic photosensitive retinal ganglion cells or melanopsin-expressing RGCs (m^+^RGCs) survive better after OHT in rats [39,40] whereas other reports indicate the contrary for mice [41] and rats [37,42,43], we have also investigated the effects of OHT in the population of m^+^RGCs. RGCs were retrogradely labelled with OHSt applied to both SCi and double immunodetected with melanopsin and Brn3a, an approach that allows to study in parallel but independently the general RGC population (Brn3a^+^) and m^+^RGCs [44]. Glaucoma is no longer considered a sole disease of the RGC population and their axons, but it also implies synaptically linked nuclei of the main primary visual pathway [13,28,45], thus the effects of ocular hypertension on the major retinal output, that is the retino-tectal projection was also investigated, for short and long periods of time after laser photocauterization of these vessels. (Short accounts of this work have been reported in Abstract format, [46,47]).

## Material and Methods

### Animal handling

All experiments were carried out following the Spanish and European Union regulations for the use of animals in research (Council Directive 86/609/EEC) and the ARVO statement for the use of animals in ophthalmic and vision research. This study was approved by the Ethics Committee for Animal Research of the University of Murcia (Spain). Adult male pigmented C57BL/6 mice (25–35g) were obtained from the University of Murcia breeding colony and were housed in temperature and light controlled rooms (12 h light/dark cycle) with food and water “ad libitum”. All surgical manipulations were carried out under general anesthesia induced with an intraperitoneal (i.p.) injection of a mixture of ketamine (70 mg/kg, Imalgene, Merial Laboratorios, S.A., Barcelona, Spain) and xylazine (10 mg/kg, Rompún, Bayer, S.A., Barcelona, Spain). While recovering from anesthesia, an ocular ointment (Tobrex, Alcon Cusí S.A. Barcelona, Spain) was applied on the cornea to prevent corneal desiccation. All efforts were taken to minimize animal suffering and analgesics were administrated during the first week. Animals were sacrificed with an i.p. injection of an overdose of pentobarbital (Dolethal Vetoquinol, Especialidades Veterinarias S.A., Alcobendas, Madrid, Spain). Experimental design is detailed in Table 1.

**Table 1:** Experimental design.

	Number of mice analyzed	Time after Laser Photocoagulation
OHSt, Brn3a and mRGCs	14	2, 4 weeks
Identification of GCL nuclei	8	4 weeks
Anterograde axonal transport	44	Naïve, 10 days, 8 or 14 weeks

Both retinas from each mouse were studied, the left treated ones, and their right contralateral to the lesion, which were used as control.

### Induction of OHT

To elevate the IOP, the left eyes were treated in a single session with diode laser burns (Viridis Ophthalmic Photocoagulator-532 nm laser, Quantel Medical, Clermont-Ferrand, France) following a modified protocol previously described that is standard in the laboratory [22,23,35,48]. The laser beam was directed, without any lenses, to the limbal and episcleral veins. The pupil of the treated eye was dilated with 1% Tropicamide (Colircusi tropicamida 1%; Alcon-Cusí, S.A., El Masnou, Barcelona, Spain) and an average of 135 spots were given per eye. The spot size, duration, and power were 50–100 μm, 0.05 s, and 0.1 W, respectively. The fellow right eyes were not treated and thus were used as control.

### Measurement of the intraocular pressure

The intraocular pressure (IOP) of both eyes was measured under anesthesia using a mice adapted rebound tonometer (Tono-Lab; Tiolat, OY, Helsinki, Finland). The readings were obtained before laser photocoagulation (LP) and at different times after LP. Only mice with a peak of IOP greater than 25 mmHg in the first 48 hours after LP were included in the study.

### Retrograde labelling from both superior colliculi

To identify RGCs with an active retrograde axonal transport a Fluorogold analogue, (OHSt) (Molecular Probes, Leiden, The Netherlands) was applied to both superior colliculi (SCi) one week before sacrifice following standard protocols previously described [44,49–55].

### Anterograde labelling of retinal afferents

To identify the retinofugal projection, four days before sacrifice, 2.5 μl of the orthogradely transported tracer cholera toxin subunit beta (CTB) were intravitreally injected (1%, diluted in distilled water, List Biological Laboratories, Campbell, CA, USA) following previously described protocols that are standard in our Laboratory [56–61].

### Tissue processing

Mice were deeply anesthetized and perfused transcardially with saline and 4% paraformaldehyde (PFA) in 0.1 M phosphate buffer. Unless otherwise stated, all products were from Sigma-Aldrich, Alcobendas, Spain.

#### Retinal wholemounts

The eyes were enucleated and both retinas were dissected and prepared as flattened whole-mounts maintaining the retinal orientation by making four radial cuts (the deepest in the superior pole) as previously described in detail [49,51,52,62,63].

#### Superior colliculi serial sections

The brains were dissected and post-fixed overnight in 4% PFA at 4°C overnight, and then cryoprotected in increasing concentrations of sucrose before embedding them in optimal cutting temperature (OCT) compound (Sakura Finetek, Torrance, CA). Serial coronal sections (30 μm) from the level of the anterior thalamus to the rostral pole of the cerebellum were obtained on a freezing cryostate.

### Immunodetection of the retinal afferents in the superior colliculi

Transported CTB from the retina to the terminals in the superior colliculi was immunolocalized using previously described methods [56–61,64]. In brief, after quenching the endogenous peroxidase, free floating sections were incubated in a solution containing goat anti-CTB antibody in PB, 2% normal rabbit serum, 2.5% bovine serum albumin and 2% Triton X-100 during 4 days at 4°C. Binding of primary antibody was visualized by incubating with biotinylated rabbit anti-goat antibody in 2% NRS, 2.5% BSA and 2% Triton X-100 in PB for 1 h at room temperature, followed by an incubation in avidin-biotin peroxidase complex (Vectastain ABC Kit Elite; Vector Laboratories, Burlingame, CA) diluted 1:100 in PB for 1 h; the peroxidase was detected using 0.025% 3,3′-diaminobenzidine tetrahydrochloride as a chromogen. After 5 min, 0.004% H_2_O_2_ was added to the solution, and 3 min were allowed for development. Sections were rinsed thoroughly in PB at 4°C and then serially ordered and mounted on gelatinized slides, air-dried, dehydrated in a series of alcohols, defatted in xylene and coverslipped with DePeX.

### Retinal immunohistofluorescence

Immunodetection on flat mounted retinas was carried out as previously described [44,51,52,65–67].

### Antibodies and working dilutions

#### Primary antibodies

see Table 2

**Table 2:** Primary antibodies used in this work.

Detection of	Molecular marker	Antibody	Source	Working dilution
RGCs	Brn3a (Pou4f1)	Goat anti-Brn3a (C-20)	Santa Cruz Biotechnologies (Heidelberg, Germany) sc-31984	1:750
m^+^RGCs	Melanopsin	Rabbit anti-melanopsin (N-terminal)	Advanced Targeting Systems (San Diego, California USA) AB-N38	1:500
Anterograde tracing	Cholera Toxin B subunit	Goat anti-CTB	List Biological Laboratories (QuadraTech, Surrey, UK) 703	1:4000

#### Secondary antibodies

Fluorescence: Donkey anti-goat Alexa 594, donkey anti-rabbit Alexa 488, (Molecular Probes, ThermoFisher, Madrid, Spain). All were used at 1:500 dilution. Histochemistry: Rabbit anti-goat IgG-biotin (1:200, Vector Laboratories, USA).

### Identification of nuclei in the ganglion cell layer

In flat mounted retinas immunoreacted against Brn3a, all nuclei of the GCL were counterstained with DAPI (4',6-diamidino-2-phenylindole, Vectashield Mounting medium with DAPI, Vector Laboratories, Burlingame, CA).

### Image acquisition

Retinas were photographed following standard procedures in our laboratory [44,49,51,52], using an epifluorescence microscope (Axioscop 2 Plus; Zeiss Mikroskopie, Jena, Germany) equipped with a computer-driven motorized stage (ProScan H128 Series; Prior Scientific Instruments, Cambridge, UK) controlled by image analysis software (IPP, Image-Pro Plus, IPP 5.1 for Windows; Media Cybernetics, Silver Spring, MD). Each reconstructed wholemount is a compound of 140 individual frames captured side-by-side with no gap or overlap between them with a 20X objective (Plan-Neofluar, 20/0.50; Zeiss Mikroskopie, Jena, Germany). When required, images were further processed using a graphics editing program (Adobe Photoshop CS 8.0.1; Adobe Systems, Inc., San Jose, CA). SCi were photographed under transmitted light, with a 5x objective to capture the whole contralateral SC in a single frame.

### Automated quantification and spatial distribution of OHSt^+^RGCs, Brn3a^+^RGCs and DAPI^+^GCL nuclei

OHSt^+^RGCs and Brn3a^+^RGCs were automatically quantified following previously described methods that are standard in our laboratory [21,49,51,52,54,55,66,67]. Briefly, the individual fluorescent images taken for each retinal frame were processed by a specific subroutine using the IPP macro language. The topology of Brn3a^+^RGCs and GCL nuclei was analyzed with isodensity maps constructed through a quadrant analysis as previously described in detail [21,44,48,49,51,52,63,68].

### Quantification and spatial distribution of melanopsin^+^RGCs

Total numbers of m^+^RGCs were obtained in the same retinas analysed 2 or 4 weeks after LP-induction of OHT. m^+^RGCs were dotted manually on the retinal photomontages. Then, dots were automatically quantified and their retinal position extracted using the IPP macro language following previously described method [44,54,65,67]. In brief, after marking the optic nerve as a reference point and drawing the retinal contour, the number of dots representing m^+^RGCs and their x, y position with respect to the optic nerve were calculated with a specific routine using the IPP macro language, these data were stored in a data sheet.

m^+^RGCs distribution was represented by nearest-neighbour maps, that were performed by a Java (Oracle Corporation, Redwood Shores, CA) application, as described [44,54,65,67]. Briefly, the user fixed the radius of the study (0.165mm) and imported the previously obtained spread sheet. Those cells within the fixed radius were counted as neighbours. Spatial information was used to plot every m^+^RGC, and each m^+^RGC was coloured within a scale from purple (0 neighbours), to red (11 or more neighbours). All plots were performed using SigmaPlot (SigmaPlot 9.0 for Windows; Systat Software, Inc., Richmond, CA).

### Volumetric analysis of retinal innervation of the visual layers in the superior colliculus

Using the image analysis software IPP, the area occupied with CTB labelling in the two most superficial layers of the contralateral superior colliculus (SC), the stratum zonale and stratum griseum superficiale, was measured by a specific IPP macro as reported [58]. A polynomial regression line (order 5; with an *r* ^2^ > 0.78 in all cases studied) was obtained for each individual SC and the integral of the function yielded the volume of the SC occupied by intense CTB labeling in each animal as previously described in detail [58,59]. This mathematical analysis allowed to calculate the volume of the SC positive for CTB signal, even in the few instances in which artefacts associated with histological mounting (e.g., wrinkles, tears, folds, and tissue debris) made few serial sections unusable for measurement. Measurements were imported into a spreadsheet (Microsoft Office Excel 2007; Microsoft Corporation, Redmond, WA) for computation and graphing.

### Statistics

All data are presented as means with standard deviations (SD). Statistical analysis was done using SigmaStat 3.1 for Windows (SigmaStat for Windows^TM^ Version 3.11; Systat Software, Inc., Richmond, CA, USA). Kruskal—Wallis was used when comparing more than two groups and Mann—Whitney when comparing two groups only. Differences were considered significant when p<0.05.

## Results

### Ocular hypertension induced by laser photocoagulation

**Fig 1:**
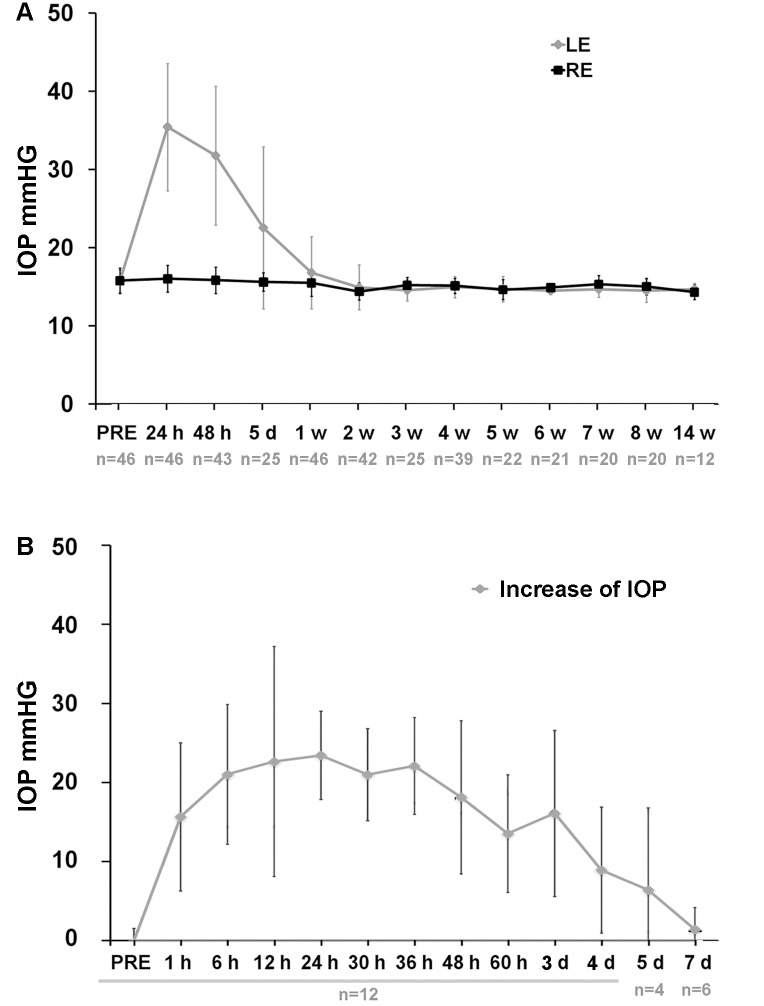
Intraocular pressure values. **A**: Graph showing the temporal course of IOP from 24h till 14 weeks after laser photocoagulation (LP). In the left eye (LE) there is a significant increase of the IOP at 24h, 48h and 5 days compared to the contralateral right eye (RE) and to prelaser (PRE) values. IOP values are back to normal from 1 week to 14 weeks, the latest time analyzed. In **B** are shown the IOP values during the first week post-LP measured at shorter intervals. One hour after LP, IOP has already increased significantly in the left eye. n = number of animals analyzed.

The intraocular pressure (IOP) values in control untreated right eyes and LP-treated left eyes are shown in detail in Fig. 1. IOP levels rise above control values during the first 5 days after LP, returned to basal levels by day 7 and remained so for up to 14 weeks, the longest survival time period analysed (Fig. 1A). Detailed readings of the IOP during the first week were analysed in a group of 12 mice used to investigate the retinotectal innervation at 14 weeks after LP. IOP increases significantly at 1 hour after LP (Mann-Whitney test, p = 0.001; n = 12), reaches maximum levels at 24 hours and remains significantly elevated until day 4 after LP (Mann-Whitney test, p = 0.004; n = 12) and then gradually return to basal values by 7 days after LP (Mann-Whitney test, p = 0.673; n = 6) (Fig. 1B).

### OHT results in focal areas lacking OHSt^+^RGCs

**Fig 2:**
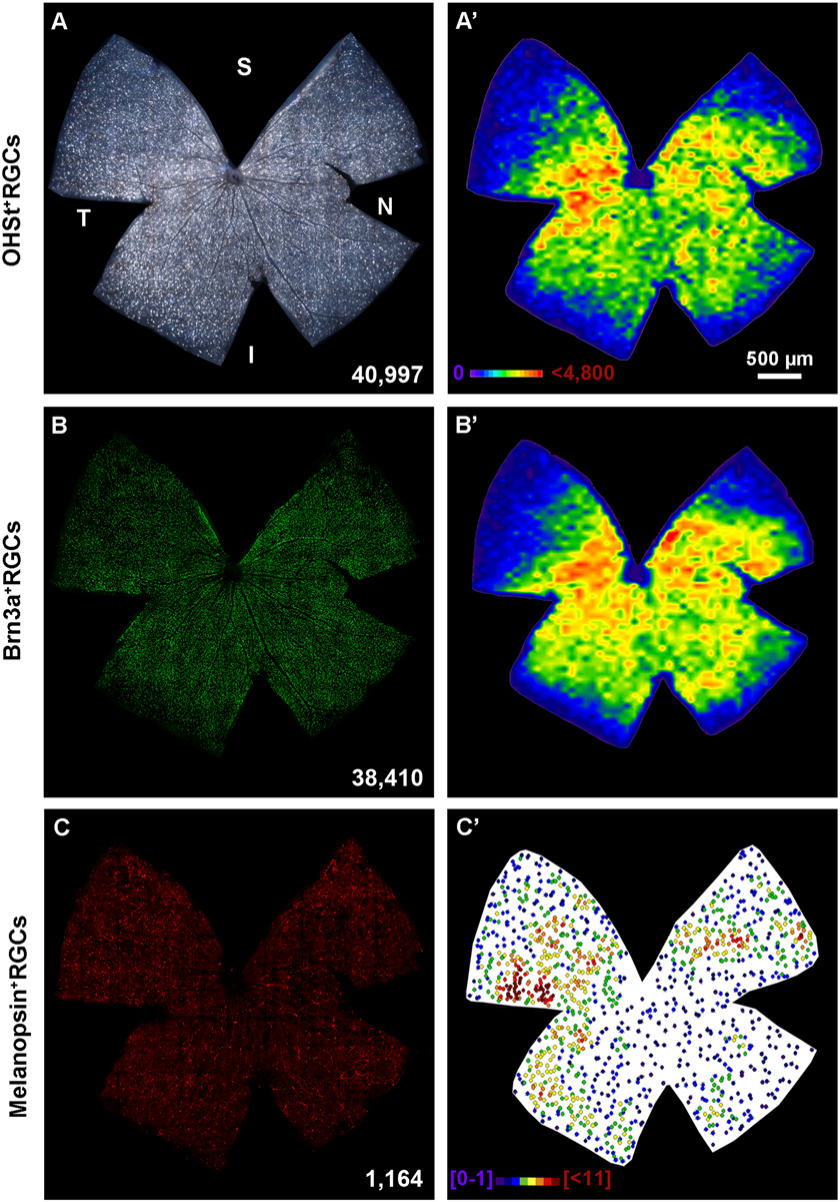
Distribution of traced-, Brn3a^+^ and melanopsin^+^ RGCs in control mice. **A-C** Photomontages from the same left retina showing OHSt (**A**), Brn3a (**B**) and melanopsin (**C**) positive RGCs. Their distribution is shown in **A'-C**', respectively. **A'-B'**: isodensity maps, **C'**: neighbour map. Density or neighbour maps colour scales in A' and C'. At the bottom of each photomontage is shown its number of RGCs or m^+^RGCs. S: superior, V: ventral, N: nasal, T: temporal. Bar: 500 μm.

In control retinas, retrogradely traced- or Brn3a^+^ RGCs were observed across the retina (Fig. 2). Their distribution is not homogeneous, rather their density is higher in the medial than in the peripheral retina (Fig. 2A', B') and their total numbers are comparable to those previously reported for pigmented mice [49,51] m^+^RGCs are found throughout the retina, although they are more abundant in the hemi- temporal and dorsal retina (Fig. 2C-C').

**Fig 3:**
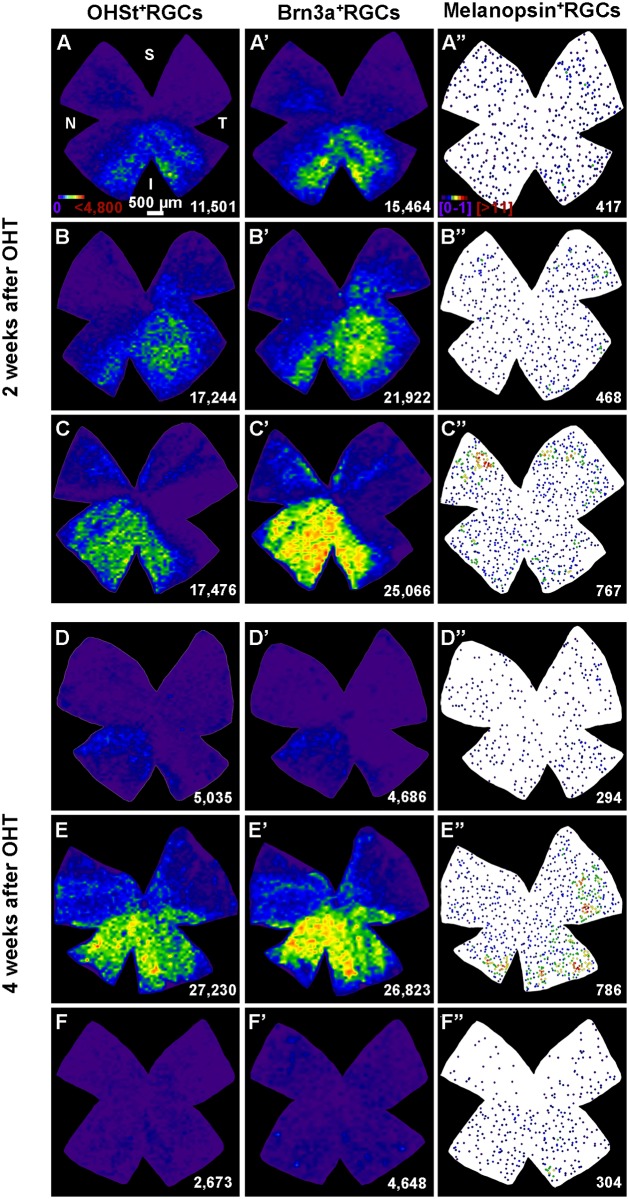
Sectorial loss of RGCs after OHT. Distribution of traced- (A-F), Brn3a^+^ (A'-F') and melanopsin^+^ (A'',F'') RGCs at 2 (**A-C''**) and 4 weeks (**D-F''**) after the induction of ocular hypertension (OHT). Maps labelled with the same letter are from the same retina (**A-A''**, **B-B''** and so on). **A-F'**: isodensity maps. **A''-F''**: neighbour maps. Colour scale for isodensity maps in A goes from purple (0 RGCs/mm^2^) to red (≥4,800 RGCs/mm^2^), and for neighbour maps in A'' goes from purple (1–2 neighbours in a radius of 0.165 mm) to red (≥11 neighbours in the same radius). At the bottom of each map is shown the number of RGCs or mRGCs counted in its respective retina. S: superior, V: ventral, N: nasal, T: temporal. Bar scale in A: 500 μm.

LP-induced OHT results in the lack of retrogradely labelled RGCs. There were large sectors with few to none retrogradely traced RGCs. The lack of OHSt^+^RGCs was often located in the dorsal retina but was also observed in the inferior retina, and varied from a small pie-shaped sector to one or several retinal quadrants (Fig. 3A-F). The lack of OHSt^+^RGCs was observed at two weeks, the earliest time point examined, and adopt the form of a pie-shaped triangular sector with their base located in the periphery and their apex in the optic disc. This lack of retrogradely labelled RGCs did not seem to progress from 2 to 4 weeks (Fig. 3D-F), since the total numbers of OHSt^+^RGCs counted in LP retinas examined at 2 or 4 weeks were comparable (Mann-Whitney test, p = 0.535). The distribution of OHSt^+^RGCs was investigated by constructing isodensity maps for each retina. These isodensity maps revealed that in addition to focal loss there was also diffuse loss of these neurons; in the LP retinas the densities of OHSt^+^RGCs are lower than those found in corresponding regions of contralateral fellow retinas (compare Fig. 3A-F with Fig. 2A').

When comparing the retinal distribution of Brn3a^+^RGCs, i.e. RGCs that are still alive independently of their retrograde labelling capability, with the distribution of retrogradely traced-RGCs it was evident that at 2 weeks the densities of Brn3a^+^RGCs were higher than those of OHSt^+^RGCs (Fig. 3AA’-CC'), but did not reach statistical significance (Mann-Whitney test, p = 0.329). At 4 weeks, this mismatch disappears and the retinal densities of both populations appear similar (Fig. 3DD’-FF') (Mann-Whitney test, p = 0.902).

### OHT induces a diffuse but not a sectorial loss of m^+^RGCs

The control fellow retinas showed a typical retinal distribution of m^+^RGCs with their total numbers comparable to those recently reported for pigmented mouse [44,69–71], a little over one thousand m^+^RGCs. The effects of OHT on the population of m^+^RGCs are comparable to those observed for the rest of the RGC population Brn3a^+^, in that the loss of m^+^RGC is proportionally comparable to that found for retrogradely traced RGCs (Fig. 3A''-F''). However, the loss of m^+^RGCs does not adopt the typical focal sectorial pattern, but rather their loss is more diffuse across the retina and more severe in the area of their higher density, the dorso-temporal retina. By two weeks after LP-induced OHT, approximately 40% of the total numbers of m^+^RGCs were lost, while at 4 weeks the loss amounted to approximately 54% of the original population. As was also observed in the same groups of retinas for the total numbers of Brn3a^+^RGCs and OHSt^+^RGCs, the differences in total numbers of m^+^RGCs between 2 and 4 weeks did not reach statistical significance (Mann-Whitney test, p = 0.209).

### OHT induces degeneration of RGCs with high inter-animal variability

**Fig 4:**
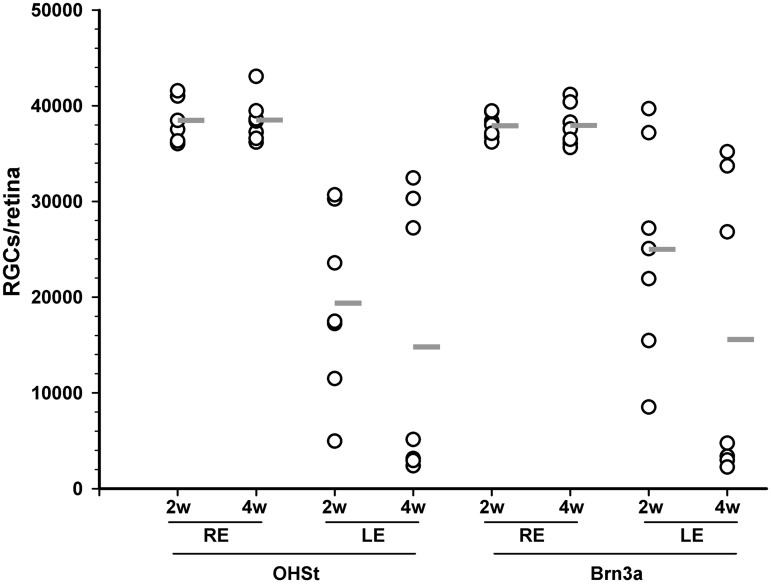
Number of RGCs in all analyzed groups. Vertical point chart showing the number of OHSt^+^RGCs (traced RGCs i.e. with a competent retrograde axonal transport) or Brn3a^+^RGCs (RGCs alive) for each of the analyzed retinas at 2 and 4 weeks after OHT (open circles). The horizontal grey line is the mean value within each group. RE: right control eye. LE: left experimental eye.

The increased IOP resulted for all experimental retinas examined in RGC loss, and the total numbers of OHSt^+^, Brn3a^+^ or melanopsin^+^ RGCs were significantly smaller when compared to their fellow right retinas. However, there was a large inter-animal variability in the extension of damage observed in the RGC population (Table 3, Fig. 4, OHSt).

**Table 3:** Total numbers of RGCs in control and OHT retinas.

Analyzed at	Population		Mean±SD
2 weeks after OHT	OHSt^+^RGCs	RE (n = 7)	38,479±2,134
		LE (n = 7)	19,383±9,499*
	Brn3a^+^RGCs	RE (n = 7)	37,904±1,265
		LE (n = 7)	25,007±11,115*
	m^+^RGCs	RE (n = 7)	1,059±79
		LE (n = 7)	629±254*
4 weeks after OHT	OHSt^+^RGCs	RE (n = 7)	38,507±2,324
		LE (n = 7)	14,795±14,326*
	Brn3a^+^RGCs	RE (n = 7)	37,936±2,151
		LE (n = 7)	15,583±15,505*
	m^+^RGCs	RE (n = 7)	1,019±140
		LE (n = 7)	478±248*

Mean ± standard deviation (SD) of the total number of traced- (OHSt^+^), Brn3a^+^ and melanopsin^+^ RGCs at 2 and 4 weeks after the induction of OHT. RE: right control retina. LE: left experimental retina. n: number of analyzed retinas.

*Significant difference when compared to right control retinas (p<0.05 Mann-Whitney test).

Two weeks after LP, there were more Brn3a^+^RGCs (25,007±11,115; n = 7) than OHSt^+^RGCs (19,383±9,499; n = 7), but this difference does not reach statistical significance probably due to the high variability of this model (Mann-Whitney test, p = 0.329) (Fig. 4, Brn3a). In accordance with the topographical analysis, at four weeks the proportion of OHSt^+^RGCs (38%) and Brn3a^+^ (41%) RGC is closer (Mann-Whitney test, p = 0.902) (Table 3, Fig. 3).

**Fig 5:**
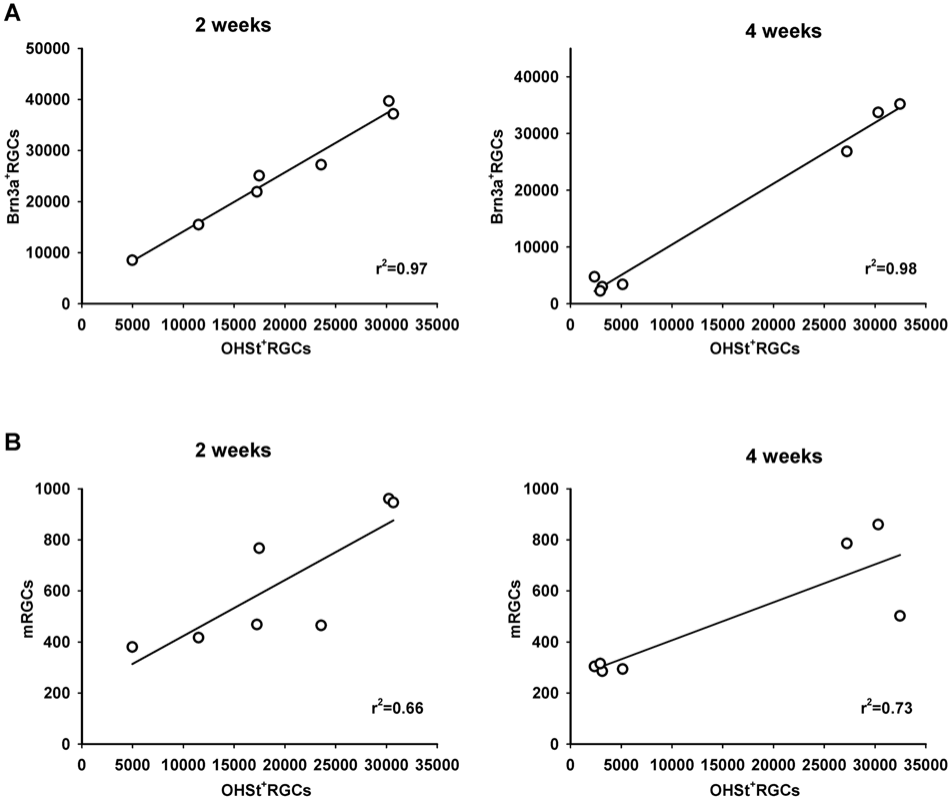
Correlation analysis. Correlation between the number of Brn3a^+^RGCs vs OHSt^+^RGCs (**A**) and mRGCs vs OHSt-RGCs (**B**) for each analyzed retina (open circles) at 2 and 4 weeks after OHT induction. The r^2^ value for each regression line is shown at the bottom right of each graph.

Correlation analysis of the number of OHSt^+^RGCs (i.e. RGCs labelled by active retrograde axonal transport) and Brn3a^+^RGCs or m^+^RGCs reveals that the loss of the Brn3a^+^RGCs is highly correlated with the extension of the axonal transport impairment (r^2^ = 0.97 at 2 weeks, and 0.98 at 4 weeks. Fig. 5A), while for the m^+^RGC population this correlation is weaker (r^2^ = 0.66 at 2 weeks and 0.73 at 4 weeks, Fig. 5B). This is in agreement with the topographical analysis (Fig. 3) where it was observed that in the areas lacking OHSt^+^RGCs there were still numerous m^+^RGCs.

Overall, we interpret these data as a suggestion that OHT results in an early alteration of the retrograde axonal transport of RGCs, as previously shown in this laboratory for albino rats [18,48] and mice [22], that occurs within the first two weeks after OHT induction and does not progress further. However, RGC loss appears to be more gradual as indicated by the progressive loss of Brn3a^+^RGCs.

### OHT induces specific loss of RGCs in the ganglion cell layer

**Fig 6:**
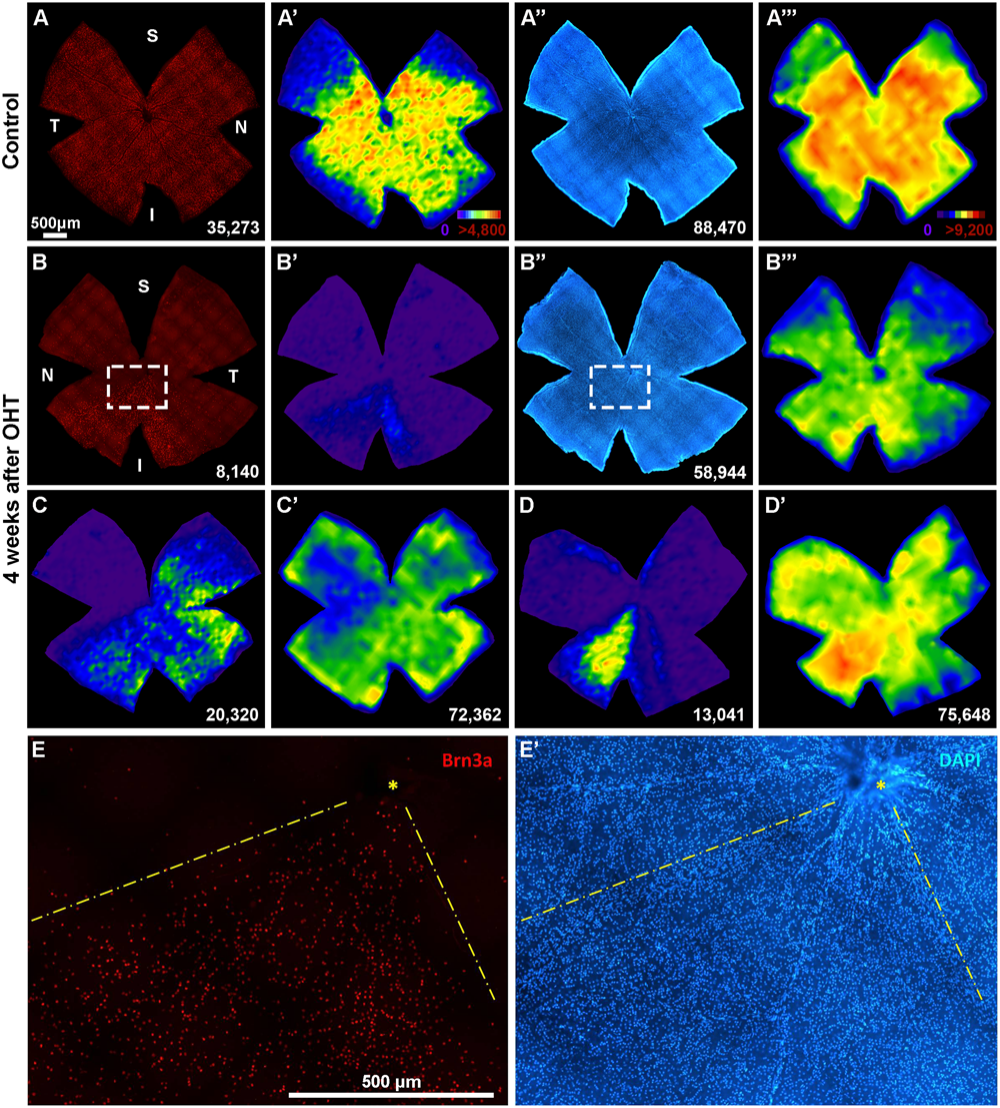
OHT does not affect non-RGC cells in the ganglion cell layer. **A,A''**, **B, B''**: retinal photomontage showing Brn3a^+^RGCs (**A,B**) or DAPI^+^nuclei in the GCL (**A'', B''**) in a control retina (**A, A''**) and in a retina analyzed 4 weeks after OHT (**B, B'**'). In **A', A'''** and **B', B'''** are shown their corresponding isodensity maps. **C,C'** and **D, D**' panels show other examples of Brn3a^+^RGCs (**C,D**) and DAPI^+^nuclei (**C',D'**) retinal distribution after 4 weeks of OHT. **E-E'**: Magnifications from the squares in B and B'' respectively showing than in the areas lacking Brn3a^+^RGCs (**E**) there are DAPI^+^nuclei (**E'**). Colour scale for Brn3a^+^RGCs isodensity maps in A' (from purple, 0 RGCs/mm^2^, to red ≥4,800 RGCs/mm^2^) and for DAPI^+^nuclei isodensity maps in A''' (from purple, 0 nuclei/mm^2^ to red ≥9,200 nuclei/mm^2^). At the bottom of each map is shown the number of RGCs or DAPI^+^nuclei counted in its respective retina. S: superior, V: ventral, N: nasal, T: temporal. Scale bar in A and B: 500 μm.

To determine whether OHT had an effect on other neurons of the GCL the total number of DAPI^+^nuclei in this layer was quantified 4 weeks after LP (Table 4, Fig. 6).

**Table 4:** Numbers of Brn3a^+^RGCs and DAPI^+^nuclei in the ganglion cell layer four weeks after OHT.

Population		Mean±SD
Brn3a^+^RGCs	RE (n = 8)	36,623±1,384
	LE (n = 8)	14,508±9,518
DAPI^+^nuclei in the GCL	RE (n = 8)	82,094±4,729
	LE (n = 8)	61,589±9,684
DAPI^+^nuclei—Brn3a^+^RGCs	RE (n = 8)	45,471±5,178
	LE (n = 8)	47,081±11,760

Mean ± SD of the total number of Brn3a^+^RGCs and of DAPI^+^nuclei in the GCL 4 weeks after the induction of OHT. The last row shows the number of DAPI^+^nuclei that are not RGCs (subtraction of Brn3a^+^RGCs from the total number of DAPI^+^nuclei). RE: right control retina, LE: left injured retina. n: number of retinas analyzed.

Four weeks after OHT around 20,000 Brn3a^+^RGCs had been lost. This proportion of cell loss is similar to the diminution in total numbers of DAPI^+^nuclei present in the GCL of the experimental retinas. Furthermore, the numbers of DAPI^+^nuclei that are not Brn3a^+^RGCs is not significantly different between the injured and the contralateral control retina (Table 4, bottom row). Overall, these data strongly suggest that at this time point, OHT does not induce the loss of other non-RGC neurons, presumably displaced amacrine cells.

The topographical analysis of the retinal distribution of Brn3a^+^RGCs and DAPI^+^nuclei in the GCL showed that in the typical pie-shaped sectors lacking Brn3a^+^RGCs there was a decrease but not an absence of DAPI^+^nuclei (Fig. 6, compare B' with B''', C with C' and D with D'). A closer look at the actual Brn3a and DAPI signals (Fig. 6E-E') shows that indeed in the areas lacking Brn3a^+^RGCs, there are many surviving cells that probably correspond mostly to displaced amacrine cells, although some of these nuclei may belong to astrocytes, endothelial and microglial cells.

### OHT affects the volume of retinal afferents innervating the visual layers of the SC

**Fig 7:**
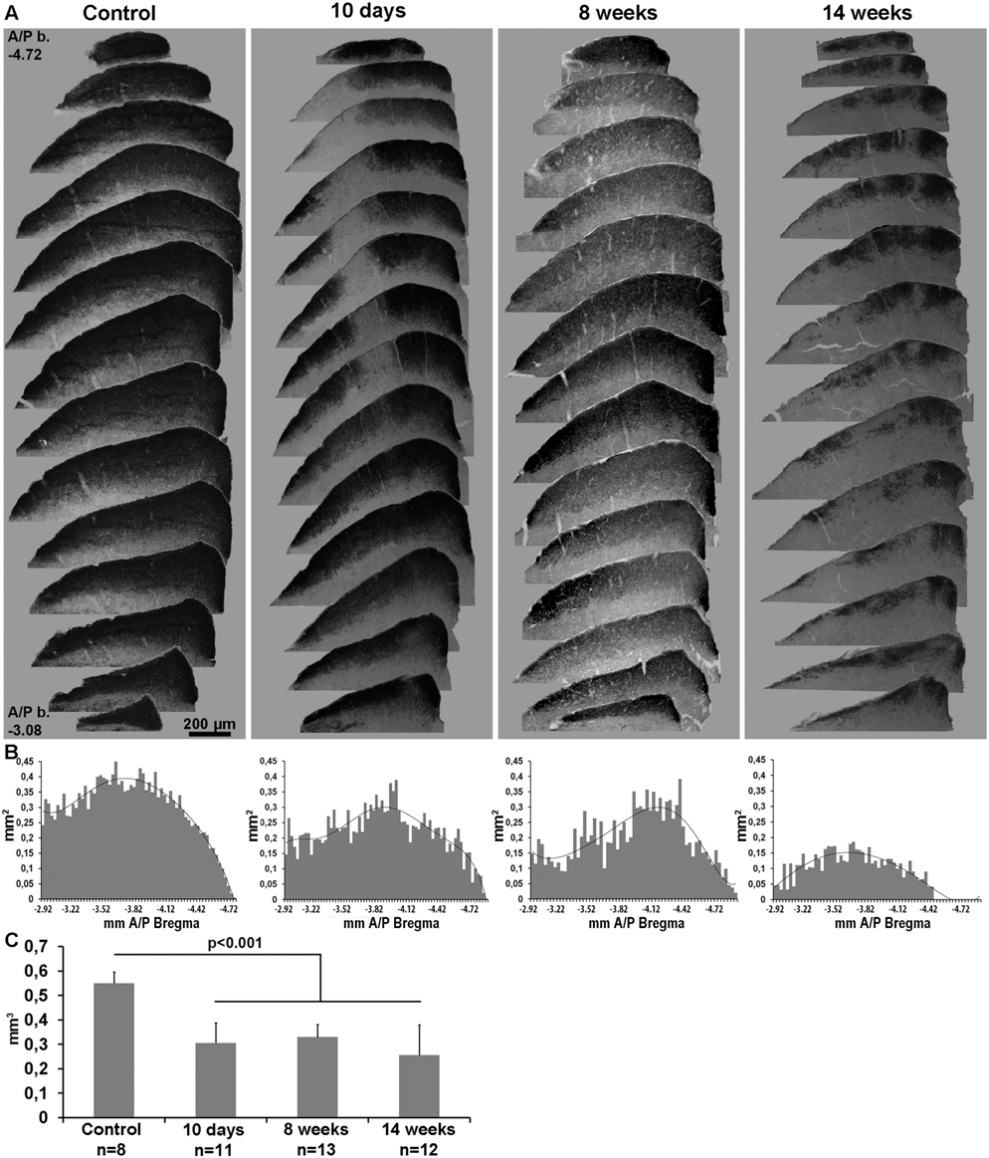
Deafferentation of the contralateral superior colliculus after OHT. **A**: Serial coronal brain sections spanning the right (contralateral) superior colliculus (from anterior/posterior bregma coordinates: -3.08 to -4.72 mm) showing the retinal afferents labelled by anterograde tracing with CTB injected into the left eye. Left column: control SC. The remaining three columns show a representative example of a SC analyzed at 10 days, 8 weeks or 14 weeks after ocular hypertension (OHT). **B**: Plots of the area occupied by retinal afferents in the SC against the anterior-posterior distance (Bregma coordinates, in mm). These plots correspond to the SCs shown in A. **C**: Volume of the SC occupied by retinal afferents. n = number of SC analyzed. Compared to control SC, the volume of SC innervated by retinal axons decreases significantly after OHT (t-test p<0.001).

We have analyzed the area and volume of the contralateral SC occupied by retinal axon terminals identified with the orthogradely transported CTB injected in the LP eye (Fig. 7). In the visual layers of control SC, the CTB labelling was homogenous through the medio-lateral and anterior/posterior Bregma coordinates. Ten days to 14 weeks after OHT induction in the left retina there was certain variability in the extent of CTB labelling throughout the right SC of individual experimental mice, but overall there was a marked reduction in the amount of CTB-labelled retinal afferents in the two most superficial layers of the contralateral SC. There were areas with almost no CTB immunoreactivity that varied in size and shape, and were present in several consecutive sections. These areas lacking CTB labelling were often restricted to small patches that extended in the dorso-ventral axis on the two most superficial visual layers; the lateral extension of these areas varied from a small narrow column to almost one fourth or one half of the medio-lateral extension of the SC, while in the rostro-caudal extension these areas were observed from few consecutive coronal sections to almost half of the rostro-caudal extension (Fig. 7). Measurement of the volume of CTB labelling in the two most superficial layers of the SC, reveals that approximately 50% (55.7% at 10 days, 59.9% at 4 weeks and 46.6% at 14 weeks) of the visual layers in the right SC do not show CTB-labelled retinal terminals. The amount of volume of the SC without CTB-labelling did not change significantly from 10 days to 14 weeks, and this is in agreement with the numbers of traced RGCs in the retina, which is stable from 15 to 30 days. Furthermore, there is a correlation between the loss of retrogradely labelled RGCs in the retina (34–50%) and the loss of CTB-labelled retinal terminals in the visual layers of the SC (46–59%).

## Discussion

Mice have been used in many experimental models of human diseases because they are easy to handle and relative inexpensive when compared to other animals, and also because of the possibility of using the transgenic technology [47]. In addition the mouse visual system has a number of advantages as well as several structural similarities to the human eye such as the outflow system and retinal vascularization [2]. One of the main difficulties in using mice relates to the small size of the eye as well as the difficulty to measure the IOP [48]. Ocular hypertension remains an important risk factor in Glaucoma and thus the development of mouse models with elevated IOP has the purpose of advancing our knowledge of the pathology of human GON, to ameliorate our understanding of the disease and to treat and prevent human GON.

Previous studies of animal models of experimental glaucoma secondary to elevated intraocular pressure have shown significant RGC loss ranging from 30 to 90% depending on the method employed, the time course and the experimental model to induce OHT in adult albino rats [16,18,21,72] and in pigmented [41] or albino [22,23] mice. It might be worth noting that in laser-induced OHT models, the loss of RGCs appears more severe in albino than in pigmented mice. Indeed, Mabuchi and colleagues (2003) [31] in pigmented mice reported a reduction of RGC axons that yields a survival at 12 weeks of approximately 30% of the original population, similar results were reported by others [29,32–34], although Yun and colleagues (2014) [36] reported a loss of approximately 60% of the RGC population by 24 weeks. However the loss of Brn3b^+^RGCs and RGC axons reported by Fu and Sretavan (2010) [24] for the albino mice at 4 weeks were of approximately 90% and 78%, respectively. Our own studies also support this idea; the present studies in pigmented mice show a loss of RGCs (50%) that is also somewhat smaller than that found in similarly treated adult albino mice (80%) [22,23]. It is possible that such a smaller RGC loss is related to the lower levels of IOP achieved in the pigmented vs albino mice after laser-induced OHT. In the present LP model, the IOP values were rather high and short-lasted (rising of 20 mm Hg for a few days) and this may be regarded as a disadvantage when compared to more chronic models of OHT that result in a slower progression of RGC loss. The elevated IOP values observed in our model are in contrast with a less dramatic increase of the IOP (rising of 6 to 7 mm Hg) that last for over one month, observed in other mouse models in which microbeads were injected into the anterior chamber to occlude aqueous outflow [5,7,13]. Nevertheless our model induces a transient OHT that is sufficient to trigger a number of features such as; the sectorial loss of RGCs, an early damage to axons at the level of the optic nerve head, the survival of RGCs with a compromised anterograde and retrograde axonal transport, all of which are typically observed in a congenic mouse model of glaucoma, the DBA/2J [10,12,13,41].

Previous studies have indicated that in experimental and human glaucoma there are important changes in the major retinorecipient target nuclei of the brain [45,73,74]. Thus, it was important to investigate the effects of ocular hypertension on the major retinofugal projection short and long periods of time after laser photocauterization. In adult albino rats Drouyer and colleagues (2008) [37] found a reduction in retinal fiber density in different retinorecipient structures with a range from approximately 50% in the vLGN to 72% in the SCN, and 50% in the SC [37]. Our results using a very sensitive orthrogradely transported neuronal tracers in adult pigmented mice are consistent with those found in adult albino rats, since we also found a lack of anterograde labelling of retinotectal terminals. Interestingly, the lack of CTB-labelled retinal terminals in the contralateral SC did not seem to progress between 10 days and 14 weeks. This finding is in concordance with previous observations in albino [22] and the present work in pigmented mice, indicating that the lack of retrogradely labelled RGCs does not progress further from 15 to 30 days. Such a lack of CTB-labelled retinotectal terminals could be due to the death of the parent RGCs and the consequent degeneration of their axons but could also be explained by an impairment of anterograde axonal transport which would be predegenerative as has been shown in a congenic adult mouse model of glaucoma [3,13,75]. Overall these results further strengthen the idea that OHT results, not only in marked degeneration of the RGC layer but also affects retinofugal axons and thus may result in significant denervation of the retinorecipient target nuclei in the brain [45,28].

The population of RGCs constitutes only a proportion of the neurons in the ganglion cell layer of the rodent [76,77]. An estimate of the actual proportion of RGCs for adult pigmented (C57BL/6) is of approximately 41% [78] or 50% [79]. In our experiments, it is likely that most of the DAPI^+^nuclei in the sectors of the retina showing a complete absence of Brn3a^+^RGCs correspond to displaced amacrine cells, with a minor proportion of these nuclei corresponding to astrocytes, endothelial cells and microglia which is known to respond with proliferation or cell migration [27,80,81]. Such observation argues for a selective damage to RGCs while sparing other non-RGC neurons in this layer. When the total numbers Brn3a^+^RGCs were deducted from the total numbers of DAPI^+^nuclei, the resulting subtraction yield comparable numbers of remaninig DAPI^+^nuclei, and this also argues in favour of a selective damage to RGCs while sparing displaced amacrine cells. Overall, the topographical loss of RGCs together with the presence of many non-RGC neurons (presumably displaced amacrine cells) in the GCL in accordance with previous observations in adult rats [21,82] and mice [41,83,84] after OHT, and suggests that damage to RGC axons may occur somewhere near the optic disc [48] where retinotopical arrangement is maximal [85–88], without affecting other neurons in the GCL. Recently, the loss of RGCs displaced to the inner nuclear or inner plexiform layer of the retina was also found to adopt the form of pie-shaped sectors following OHT in adult albino rats [54], suggesting that if the GCL were to be the primary site for OHT-induced damage, displaced RGCs would have been found within the areas lacking RGCs, and this was not so (See Figure 10 of Nadal-Nicolás et al., 2014 [54]).

Non-image forming visual behaviours are dependent on the intrinsically photosensitive RGCs containing melanopsin (mRGCs) and include photoentrainment of circadian cycles, photic suppression of activity, acute light-activated suppression of pineal melatonin secretion, and control of pupillary light responses [44,52,71,89–91]. In Human GON patients, there were significant reductions in intrinsically photosensitive RGC (ipRGC) function of the glaucomatous eye when compared to the contralateral eye or to normal populations [92–94]. A previous study has reported that mRGCs were more injury-resistant in chronic ocular hypertension model with no alterations in the total numbers nor in their dendritic morphology up to 12 weeks after OHT induction in adult albino rats [39], suggesting that m^+^RGCs carry some unique characteristics that are different from other populations of RGCs. A larger number of studies, however, indicate that this is not the case [40] for experimental glaucoma. In adult rats, laser-induced OHT results in significant reductions of the mRGCs [37,42,43] as well as in their innervation of the suprachiasmatic nuclei with an impact on their ability to entrain to light [37]. Similarly, Jakobs and colleagues (2005) [41] reported loss of m^+^RGCs in adult mice with congenic glaucoma and in a line of mice created by crossing DBA/2J mice (which develop a pigmentary glaucoma) with Thy1-CFP mice [38], the progressive increase of the IOP was accompanied by a concomitant reduction in the numbers of RGCs and of m^+^RGCs, suggesting that OHT-induced progressive loss of RGCs, and of m^+^RGCs, could have a substantial impact on animal behavioural response patterns (Zhang et al., 2013). Our present results in pigmented mice also indicate that m^+^RGCs degenerate as a consequence of OHT (Fig. 3; Table 3). The retinas analyzed 2 or 4 weeks after LP were examined for the presence of Brn3a^+^RGCs and m^+^RGCs to ascertain the fate of the general population of RGCs (Brn3a^+^RGCs) and the subpopulation of intrinsically photosensitive RGCs (m^+^RGCs). Thus the proportions of surviving m^+^RGCs and Brn3a^+^RGCs found at 2 or 4 weeks are comparable, since the retinas were exposed to the same amount of elevated IOP. Approximately 40% to 54% of the original m^+^RGCs were lost at 2 or 4 weeks after LP, and these proportions were comparable among themselves and to those observed for Brn3a^+^RGCs at similar time intervals, indicating that OHT also induces the loss of m^+^RGCs in similar proportions. Our results however, demonstrate that the loss of m^+^RGCs is rather diffuse and does not follow the typical pattern of pie-shaped sectors observed for the general population of Brn3a^+^RGCs. Possible explanations for these discrepancies may be related to the types of m^+^RGCs analyzed in different studies (there are 6 different morphological types of m^+^RGCs; [40]). Nevertheless, the possibility that m^+^RGCs are more resistant to axotomy [95,96], NMDA-induced excitotoxicitiy [97] and mitochondrial optic neuropathies [98,99] than the rest of the RGC population remains to be further studied [40].

In the present study we have further characterized the course of retinal degeneration after laser-induced OHT. To this end we have used: neuronal tracers to assess both the retrograde and anterograde transport; specific molecular markers such as Brn3a antibodies to identify all RGCs except melanopsin containing RGCs, and melanopsin antibodies to detect ipRGCs (M1 to M4, but no M5), and; nuclear staining to detect and quantify non-RGC neurons in the ganglion cell layer of the retina. We show that at 2 weeks after LP, there are still surviving RGCs whose axonal transport is compromised and results at 4 weeks in their loss. These data are relevant when designing neuroprotective treatments. We have also investigated the intrinsically photosensitive subtype of RGCs, the m^+^RGCs, because in glaucoma patients the circadian rhythm and/or the pupil response are altered [92–94], and show that while m^+^RGCs are numerically affected in the same proportion as the rest of RGCs their topographic loss does not follow the rest of the RGC population. Finally, long-time after LP the main retinorecipient target nuclei in the brain, the SC, shows that the volume occupied by retinotectal afferents is reduced to approximately half their normal values.
